# Metabolomics and biochemical alterations caused by pleiotrophin in the 6-hydroxydopamine mouse model of Parkinson’s disease

**DOI:** 10.1038/s41598-022-07419-6

**Published:** 2022-03-04

**Authors:** Esther Gramage, Jorge Sáiz, Rosalía Fernández-Calle, Yasmina B. Martín, María Uribarri, Marcel Ferrer-Alcón, Coral Barbas, Gonzalo Herradón

**Affiliations:** 1 Departamento de Ciencias Farmacéuticas y de la Salud, Facultad de Farmacia, Universidad San Pablo-CEU, CEU Universities, Urbanización Montepríncipe, 28660 Boadilla del Monte, Madrid Spain; 2 Centre for Metabolomics and Bioanalysis (CEMBIO), Department of Chemistry and Biochemistry, Facultad de Farmacia, Universidad San Pablo-CEU, CEU Universities, Urbanización Montepríncipe, 28660 Boadilla del Monte, Madrid Spain; 3 Departamento de Anatomía, Facultad de Medicina, Universidad Francisco de Vitoria, Ctra. Pozuelo-Majadahonda KM 1.800, 28223 Pozuelo de Alarcón, Madrid Spain; 4 BRAINco Biopharma, S.L., Bizkaia Technology Park, Zamudio, Spain

**Keywords:** Parkinson's disease, Neurotrophic factors

## Abstract

Pleiotrophin (PTN) is a cytokine involved in nerve tissue repair processes, neuroinflammation and neuronal survival. PTN expression levels are upregulated in the nigrostriatal pathway of Parkinson’s Disease (PD) patients. We aimed to characterize the dopaminergic injury and glial responses in the nigrostriatal pathway of mice with transgenic *Ptn* overexpression in the brain (*Ptn*-Tg) after intrastriatal injection of the catecholaminergic toxic 6-hydroxydopamine (6-OHDA) at a low dose (5 µg). Ten days after surgery, the injection of 6-OHDA induced a significant decrease of the number of tyrosine hydroxylase (TH)-positive neurons in the substantia nigra and of the striatal TH contents in Wild type (Wt) mice. In contrast, these effects of 6-OHDA were absent in *Ptn*-Tg mice. When the striatal Iba1 and GFAP immunoreactivity was studied, no statistical differences were found between vehicle-injected Wt and *Ptn*-Tg mice. Furthermore, 6-OHDA did not cause robust glial responses neither on Wt or *Ptn*-Tg mice 10 days after injections. In metabolomics studies, we detected interesting metabolites that significantly discriminate the more injured 6-OHDA-injected Wt striatum and the more protected 6-OHDA-injected *Ptn*-Tg striatum. Particularly, we detected groups of metabolites, mostly corresponding to phospholipids, whose trends were opposite in both groups. In summary, the data confirm lower 6-OHDA-induced decreases of TH contents in the nigrostriatal pathway of *Ptn*-Tg mice, suggesting a neuroprotective effect of brain PTN overexpression in this mouse model of PD. New lipid-related PD drug candidates emerge from this study and the data presented here support the increasingly recognized “lipid cascade” in PD.

## Introduction

Parkinson’s disease (PD) is a neurodegenerative disorder that is characterized by the progressive loss of dopaminergic neurons in the substantia nigra (SN) and dopaminergic terminals of the striatum. The underlying cause of this neuronal loss is not completely understood. Neuroinflammation, characterized by excessive microgliosis and astrogliosis, has been implicated in the pathophysiology of PD, being critical to PD progression^1,2^. Thus, identification of the molecular mechanisms involved in dopaminergic cell survival and modulation of the neuroimmune response represents an opportunity to discover novel therapeutic targets and/or biomarkers in PD. Pleiotrophin (PTN) is one neurotrophic factor recently shown to be an important regulator of neuroinflammation in different pathological contexts^3^.

Pleiotrophin is a survival factor for dopaminergic neurons and promotes tyrosine hydroxylase (TH) expression^4^. PTN is also a survival factor for the catecholaminergic PC12 cell line^5^. Genetic inactivation of *Ptn* exacerbates amphetamine-induced dopaminergic injury in the nigrostriatal pathway^6^, including the unexpected loss of 20% of dopaminergic neurons in the SN that is not observed in amphetamine-treated Wild type (Wt) mice. Interestingly, amphetamine- and cocaine-induced alterations in the striatal phosphoproteome of *Ptn*^−/−^ mice were similar to those found in PD^7–9^, suggesting that PTN may protect against neuronal injury in different brain disorders. PTN is one of the factors upregulated after treatment with levodopa in the denervated striatum of parkinsonian rats^10,11^, suggesting a role for this cytokine in the neurotrophic mechanisms and plasticity triggered by treatment with levodopa. Interestingly, PTN is upregulated in the dorsolateral striatum in the 6-hydroxydopamine (6-OHDA) rat model of PD, suggesting neurotrophic actions of this cytokine in this model^12^. Supporting this, PTN is involved in the cAMP-dependent enhancement of the differentiation of dopaminergic neurons in cultures^13^, prevents amphetamine- and cocaine-induced toxicity in PC12 cells^5,14^ and exerts trophic effects on donor cells after neural transplantation in vivo to achieve functional recovery of nigrostriatal pathways in animal models of PD^15^. Furthermore, striatal and/or nigral PTN over-expression provides neuroprotection against the dopaminergic toxic 6-OHDA in rats^16,17^.

The proven neuroprotective effects of PTN in different models are interesting because *Ptn* expression is upregulated in the brain in different pathologies such as PD, Alzheimer’s disease (AD), ischemia, and after administration of different drugs of abuse including amphetamine and alcohol^3^. In PD patients, PTN is upregulated in the degenerating substantia nigra, apparently in dopaminergic neurons with downregulated expression of TH^18^. Recently, loss of pericytes in PD, AD, amyotrophic lateral sclerosis (ALS), human immunodeficiency virus (HIV)-associated neurocognitive disorder and Huntington’s disease has been linked to PTN depletion causing neuronal loss^19^. These different disorders are characterized by overt neuroinflammation and PTN has been recently found to modulate the neuroimmune response in different pathological contexts. For instance, transgenic *Ptn* overexpression in the brain potentiates striatal astrocytosis induced by acute administrations of amphetamine^20^. *Ptn* overexpression also potentiates microglial activation and increases of pro-inflammatory cytokines in the brain induced by an acute administration of lipopolysaccharide (LPS)^21^.

The evidence summarized here suggests that PTN potentiates the acute neuroimmune response induced by different stimuli, including microglial and astrocytic responses, which is necessary and critical for host defence^22^. However, persistent and/or over-activation of microglia is deleterious. Thus, one aim of the present work was to shed some light on the possible role of PTN in the subchronic nigrostriatal neuroinflammation associated with the 6-OHDA mouse model of PD. In addition, understanding altered metabolic pathways and metabolites involved in the development and progression of disease provides a better knowledge of the underlying related biological alterations. This is important for diseases like PD, for which the biomolecular causes are still unclear and, of particular interest in early stages of the disease when neuroprotective strategies are expected to be more successful. In these regards, metabolomics has become an important tool able to provide useful insights into unknown biochemical mechanisms and possible biomarkers for various disorders. For that reason, we aimed to confirm if *Ptn* overexpression in the mouse brain prevents dopaminergic injury in an 6-OHDA model with a partial lesion and how PTN modifies biochemical cascades, using an untargeted metabolomic approach.

## Methods

### Animals

*Ptn*-Tg mice on a C57BL/6J background were generated by pronuclear injection as previously described^23,24^. PTN specific overexpression in different brain areas, including a ~ 20% upregulation in the striatum, was established previously^9^.

We used male *Ptn*-Tg and Wt animals of 9–10 weeks (20–25 g). Mice were housed under controlled environmental conditions (22 ± 1 °C and a 12 h light/12 h dark cycle) with free access to food and water.

All the animals used in this study were maintained in accordance with European Union Laboratory Animal Care Rules (2010/63/EU directive) and protocols were approved by the Animal Research Committee of USP-CEU (authorization reference: PROEX 86/14). Animal studies were carried out in compliance with the ARRIVE guidelines.

### 6-OHDA lesion

All surgical procedures were conducted under general anesthesia induced with an intraperitoneal mixture of ketamine (0.075 mg/g)–xylazine (0.02 mg/g). Anesthetized animals were placed into the stereotaxic frame (Kopf Instruments, CA, USA), and 6-OHDA hydrochloride (Sigma-Aldrich, Madrid, Spain) was injected into the right striatum according to the coordinates anterior–posterior: + 0.5; medial–lateral: + 0.21; dorsal–ventral: 3.0 relative to bregma^25^. 6-OHDA was dissolved at 5 μg per 1 μL of a solution containing 0.9% NaCl and 0.02% ascorbic acid (vehicle). The experimental animals were injected with 1 μL of the 6-OHDA solution at a rate of 0.5 μL/min using a Hamilton syringe equipped with a 30-gauge stainless steel needle. The needle was withdrawn 2 min after the injection. Sham-operated animals were injected with vehicle at the same coordinates. The experimental groups included in the study are: vehicle-injected Wt, 6-OHDA-injected Wt, vehicle-injected *Ptn*-Tg and 6-OHDA-injected *Ptn*-Tg.

### Behavioural studies

Locomotor asymmetry and motor coordination were tested in 6-OHDA-treated mice. As a low dose of 6-OHDA was used and therefore gross alterations in behavioural responses were not expected, mice were not exposed to the behavioural tests until 10 days after injections, when they were evaluated. First, each mouse was tested on the cylinder test early in the morning. Two hours after that, rotarod test was performed. Mice were euthanized one hour after finishing the rotarod experiment.

#### Rotarod test

Motor coordination and balance was studied in 6-OHDA-treated mice utilizing a rotarod apparatus (Panlab/Harvard apparatus). Each animal was placed on the rod four times, in an accelerating mode starting at a rate of 4 rpm, with a 15-min interval between sessions. Animals unable to hold on to the rod for more than 1 min are excluded from the study. In the present study, all animals were able to hold on to the rod for more than 1 min, so none of them were excluded from the experiment. The maximum time of the four attempts was used for statistical analysis^26^.

#### Cylinder test

To assess locomotor asymmetry, mice were gently placed individually in a transparent plastic cylinder 10 cm in diameter and 20 cm in height. The number of wall contacts with left and right upper limbs were recorded within 10 min. A mirror was placed on the opposite side of the observer to capture all the behaviors. After the experiment, the asymmetry score was calculated by expressing the performance of the contralateral limb (left limb contacts) as a percentage of the total performance^27,28^.

### Immunohistochemical analysis

Ten days after the surgery, mice (n = 4–5/group/genotype) were sacrificed by perfusion with 4% p-formaldehyde.

Thirty-micron serial frontal sections of the midbrain in the region of the SNpc and striatal free-floating sections were processed as previously described^6,29^. Immunostaining was carried out on free-floating sections with a standard avidin–biotin immunocytochemical protocol previously described^6^. Striatal and SNpc sections were incubated overnight with rabbit TH antiserum (Chemicon International, Temecula, CA, USA) diluted 1:1000. After careful washing, the sections for TH analysis were incubated with the biotinylated secondary antisera (Vector, Burlingame, CA) at room temperature. The avidin–biotin reaction was performed using a Vectastain ABC peroxidase kit following the protocol suggested by the manufacturer. The immunoreactivity was visualized using 0.06% diaminobenzidine (Sigma-Aldrich (St Louis, USA)) and 0.03% H_2_O_2_ diluted in PBS. In addition, striatal sections were incubated overnight at 4 °C with anti-ionized calcium-binding adaptor molecule 1 (Iba1, Wako, Osaka, Japan; 1:1000) and anti-glial fibrillary acidic protein (GFAP; Millipore, Madrid, Spain; 1:1000) antibodies, followed by 30 min incubation with the Alexa-Fluor-555 and Alexa-Fluor-488 corresponding secondary antibodies (Invitrogen, Waltham, MA USA; 1:500). Photomicrographs were captured with a digital camera coupled to an optical microscope (DM5500B) and the Leica SCN400 Scan Scanner (Leica, Solms, Germany).

Quantification of expression of TH in striatum sections was performed with the aid of the Fiji image analysis system, based on Image J (NIH, Bethesda, MD, Version 1.50f)^30^. Sections were scanned and the whole striatal regions of the injected size were selected as regions of interest (ROIs). The TH immunoreactivity was measured in both hemispheres. For the image processing with Fiji, background was sustracted and image was converted to binary mode. Threshold were equally established in all the groups. Percentage of marked area was considered as the proportion of TH-immunoreactivity of the injected side (right) stablishing as a control the contralateral side (left) of the same section^31,32^. A total of 5–6 rostrocaudal sections were used per animal.

The total number of TH-positive SNpc neurons was counted in the 6-OHDA—or vehicle—injected side of each animal using the optical fractionator^32^. The researcher doing the stereological counting was blind to the experimental condition of the animal being counted. This unbiased method of cell counting is not affected by either the volume of reference (SNpc) or the size of the counted element (neurons). TH-positive neurons were counted in the right SNpc (6-OHDA or vehicle injected) of every four sections (9–12 sections per animal) throughout the entire extent of the SNpc^33^. Each midbrain section was viewed at low power (4× objective) and the SNpc was outlined by using the set of anatomic landmarks defined previously^33^. Then, starting at a random microscope visual field, the number of TH-stained cells was counted (20×). To avoid double counting of neurons with unusual shapes, TH-stained cells were counted only when their nuclei were optimally visualized which occurred only in one focal plane.

Iba1+ cells and GFAP+ astrocytes were counted in 1100 µm × 1400 µm standardized areas of three different sections in the striatum using ImageJ, following methods previously described^21,29^. ROIs were consistently selected in striatal representative areas of all the experimental groups. Total marked area for Iba1 and GFAP was calculated as overall image fluorescence, subtracting the mean background fluorescence and the number of positive cells were obtained with the "Analyze Particle" function in ImageJ. Glial cell size was determined by using the threshold and count particles features of Fiji software. Mean particle size was calculated by performing the average of the individual values of particle size of Iba-1 or GFAP positive cells. The relative cell size for each experimental group was calculated as fold change of the control group (Wt vehicle). Three sections were quantified per animal/experimental group.

### Metabolomics

#### Reagents and solvents

Methanol (MeOH) MS grade and methyl tert-butyl ether (MTBE) were obtained from Sigma–Aldrich (Steinheim, Germany). Isopropanol (IPA) was from Fischer (Austria). Ammonium hydroxide 28% was from VWR Collection Chemicals (USA). Formic acid 98% was obtained from Sigma-Aldrich (St Louis, USA). Ultra-pure water was obtained from a Milli-Q-Plus 185 system (Millipore, USA).

#### Sample preparation for metabolomics analysis

Striatum resections from wild type (Wt, 9–12/treatment) and *Ptn*-Tg mice (4–7/treatment) were added to 300 µL MeOH:water (50:50) in 2 mL Eppendorf tubes and were first homogenized with glass beads in a TissueLyser LT (QIAGEN) for 2 min. The tubes were immersed in liquid N_2_ and homogenized again in the TissueLyser LT for another 2 min. A volume 100 µL of the homogenate was transferred into Eppendorf tubes of 1.5 mL and added with 320 µL of methanol and 80 µL of MTBE and the mixture was vortexed for 1 h. Afterwards, the vials were centrifuged at 4000*g* for 20 min at 20 °C and 300 µL of the supernatants were transferred into new tubes, which were evaporated to dryness in a vacuum concentrator. Finally, the residues were reconstituted in 50 µL of MeOH:water:MTBE (37:5:4), being the samples ready for their analysis. Blank samples were prepared following the sample procedure without the addition of any biological tissue.

#### LC-ESI-qTOF/MS sample analysis

Analyses were performed in a HPLC 1200 Agilent system coupled to an Agilent 6520 qTOF mass spectrometer operated in mode full scan from 50 to 1200 m/z, with a RP C8 column Agilent Poroshell (150 mm 2.1 mm, 2.7 μm) as previously described^34^. Mobile phases were composed by 5 mmol/L NH_4_HCO_2_ in ultra-pure water (phase A) and 5 mmol/L NH_4_HCO_2_ in MeOH (85%) and IPA (15%) in phase B, in positive ionization mode, and formic acid 0.1% in ultra-pure water (phase A) and formic acid 0.1% in MeOH (85%) and IPA (15%), for the negative ionization mode, pumped at 0.5 mL/min. The capillary voltage (V) was 3500 kV in positive ionization mode and 4500 kV in negative ionization mode. Reference masses were infused in all analyses to perform the mass correction, which were 121.0509 m*/z* and 922.0098 m*/z* for the positive ionization mode and 112.9856 m*/z* and 1033.9881 m*/z* for the negative ionization mode. Data files were collected in centroid mode at a scan rate of 1.02 scans/s.

#### Data analysis

All data were controlled and acquired using Mass Hunter Qualitative Analysis B.07.00 (Agilent Technologies). Data obtained from LC–MS were cleaned of background noise and unrelated ions. Peak detection, deconvolution and alignment were performed by the recursive feature extraction (RFE) using Profinder Software B.08.00 (Agilent Technologies). Blank subtraction and filtering by frequency of at least 50% of the QC and 60% of each group and relative standard deviation (RSD) less than 30% in QC were performed, to keep only the relevant features. Missing values were substituted by KNN algorithm.

#### Metabolite annotation

Selected features were annotated based on their composite MS spectra using CEU Mass Mediator (CMM, http://ceumass.eps.uspceu.es)^35^, an online tool for aiding researchers in metabolite annotation of mass spectrometry data.

### Statistical analysis

Data from immunohistochemistry analysis and behavioural tests are presented as mean ± standard error of the mean (S.E.M.) and were analyzed using two-way ANOVA considering genotype and treatment as variants. Relevant differences were analyzed by post-hoc comparisons with Tukey’s post-hoc tests. *P* < 0.05 was considered as statistically significant. All statistical analyses were performed using Graph-Pad Prism version 8 (San Diego, CA, USA).

In the metabolomics studies, multivariate analysis as Principal Components Analysis (PCA) and Orthogonal Partial Least Square Discriminant Analysis (OPLS-DA) with Variables Importance Projection (VIP) scores and p(corr) values were performed in order to discriminate the classes. Univariate analysis was performed by t-tests P-value. A combination of the VIP Score > 1.0 and absolute values of p(corr) > 0.5 was used to primarily select relevant features. Then, a combination of p < 0.05 and the absolute value of the percentage of variation > 20% was selected for the definition of statistically significant features. Multivariate analyses were performed in SIMCA 16 (Umetrics, Umeå Sweden).

## Results

### Absence of 6-OHDA-induced loss of TH contents in the nigrostriatal pathway of *Ptn*-Tg mice

**Figure 1:**
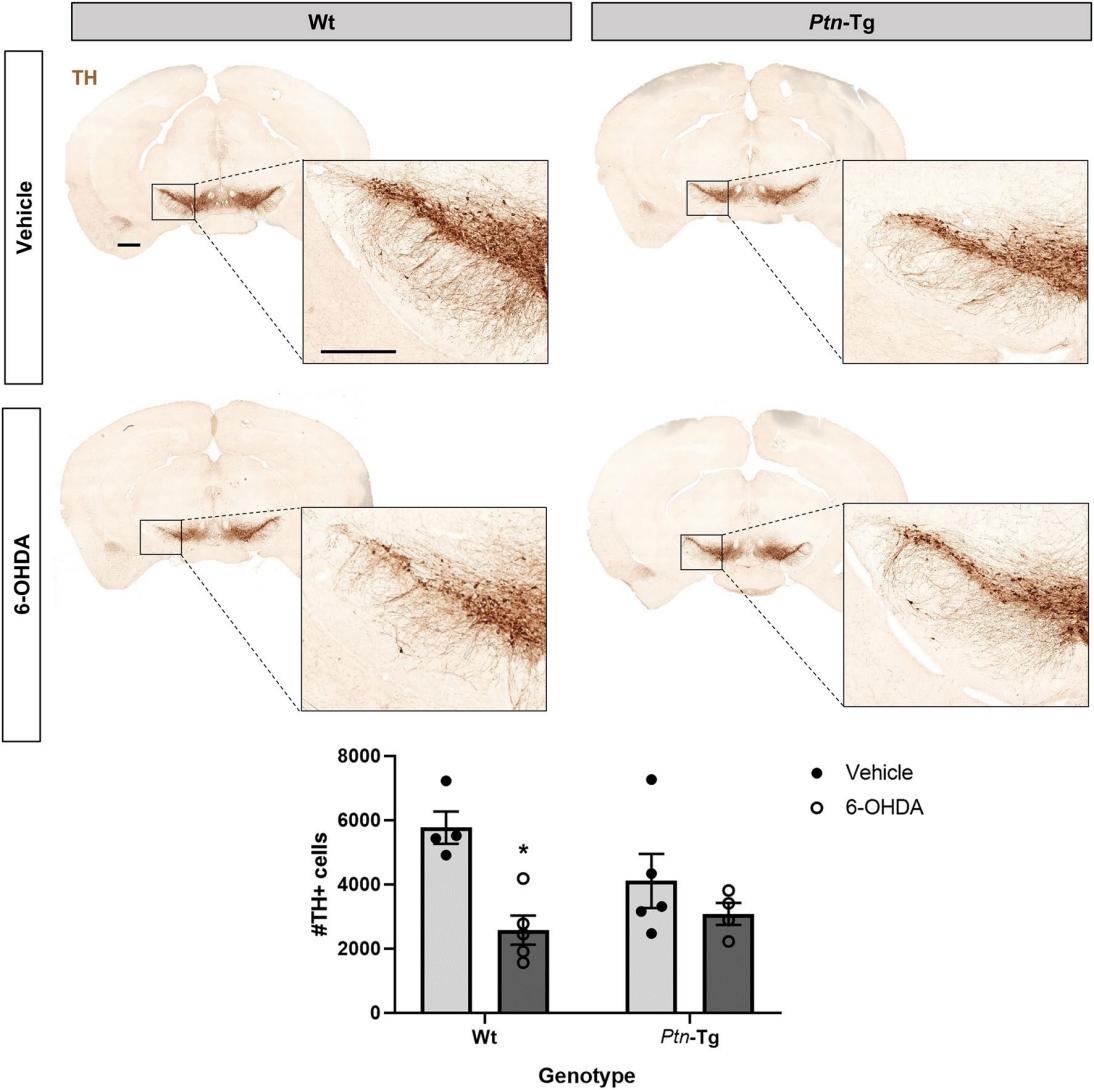
6-OHDA induces loss of TH+ neurons in the substantia nigra of Wt mice. Photomicrographs of substantia nigra of TH-immunostained sections from mice sacrified 10 days after vehicle or 6-OHDA striatal injection. Graph shows the number of TH+ cells in the substantia nigra pars compacta counted by stereology. Data are represented as mean ± S.E.M. *P < 0.05 vs. Wt-Vehicle. Scale bar = 500 μm (magnification image scale bar = 300 μm).

Before sacrifice, animals were tested for alterations in motor functions by means of rotarod and cylinder tests. We did not observe any changes in motor functions caused by the treatment or the genotype (Supplementary Fig. 1). To test the possibility that *Ptn*-Tg mice may be protected against 6-OHDA-induced neurotoxicity in the SN, we assessed the number of TH+ cells in this area of vehicle- and 6-OHDA-treated *Ptn*-Tg and Wt mice. ANOVA revealed a significant effect of the treatment (F(1,14) = 12.17; P = 0.0036). Post-hoc comparisons revealed that vehicle-treated *Ptn*-Tg mice did not show significant differences in the number of TH+ cells compared to Wt mice (Fig. 1, P = 0.25). We found a significant decrease in the number of TH+ cells in the substantia nigra of Wt mice treated with 6-OHDA compared to vehicle-treated mice (Fig. 1, P = 0.0106). In contrast, we found that 6-OHDA did not cause a significant change in the number of TH+ cells of *Ptn*-Tg mice compared with vehicle-treated animals (Fig. 1, P = 0.64). The data suggest that the severity of TH+ cell loss in the SN induced by 6-OHDA is limited by PTN.

**Figure 2:**
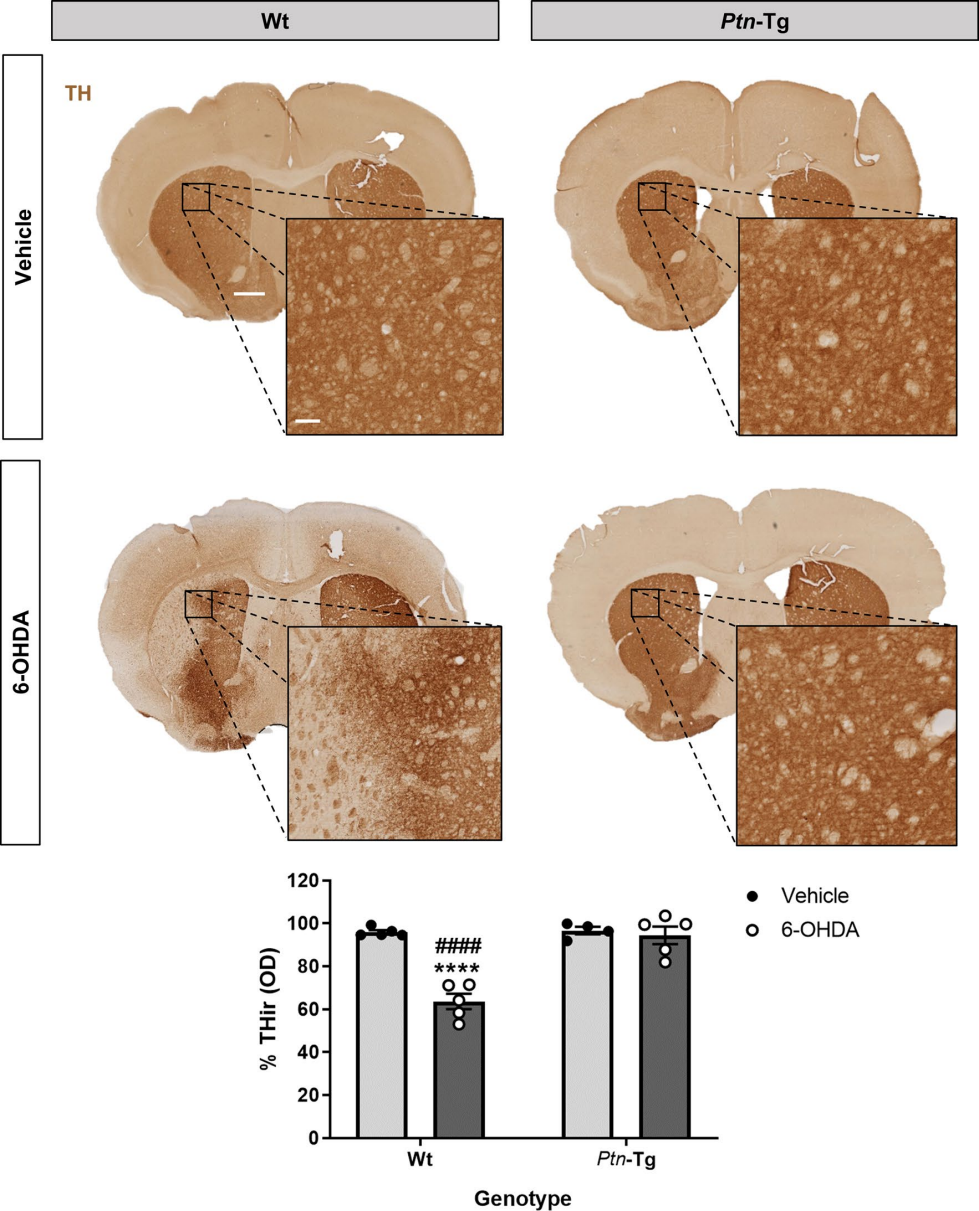
6-OHDA-induced loss of TH expression in striatum is prevented in *Ptn*-Tg mice. Photomicrographs illustrate that PTN overexpression prevents 6-OHDA-induced dopaminergic damage. TH-immunostained striatal sections of mice, 10 days after vehicle or 6-OHDA striatal injection. Graph shows the proportional stained area of TH-ir (TH-immunoreactivity) in the striatum. Data are represented as mean ± S.E.M. OD: optical density. ****P < 0.0001 vs. Wt-Vehicle. ^####^P < 0.0001 vs. *Ptn*-Tg-6-OHDA. Scale bar = 500 μm (magnification image scale bar = 100 μm).

We also analyzed in immunohistochemistry studies TH expression in the striatum of *Ptn*-Tg and Wt mice treated with 6-OHDA or vehicle (control). ANOVA revealed a significant effect of the genotype (F(1,15) = 32.37; P < 0.0001), a significant effect of the treatment (F(1,15) = 27.15; P < 0.0001) and a significant interaction genotype x treatment (F(1,15) = 24.65; P = 0.0002). Post-hoc analysis did not detect significant differences in the levels of TH in the striatum of vehicle-treated *Ptn*-Tg and Wt mice (Fig. 2, P = 0.99), suggesting that overexpression of PTN is not a key factor for TH expression in the mouse striatum in normal condition. We found that 6-OHDA caused a significant depletion of TH contents in the striatum of Wt mice compared with vehicle-treated Wt mice (Fig. 2, P < 0.0001). Interestingly, we did not observe a decrease of TH levels in the striatum of *Ptn*-Tg mice treated with 6-OHDA (Fig. 2, P = 0.96). The data confirm that 6-OHDA produces degeneration of dopaminergic terminals in the striatum of Wt mice, effect that was absent in *Ptn*-Tg mice. Overall, the data suggest that PTN modulates the severity of 6-OHDA-induced neurotoxicity in the mouse nigrostriatal pathway.

### 6-OHDA-induced glial responses in Wt and *Ptn*-Tg mice

**Figure 3:**
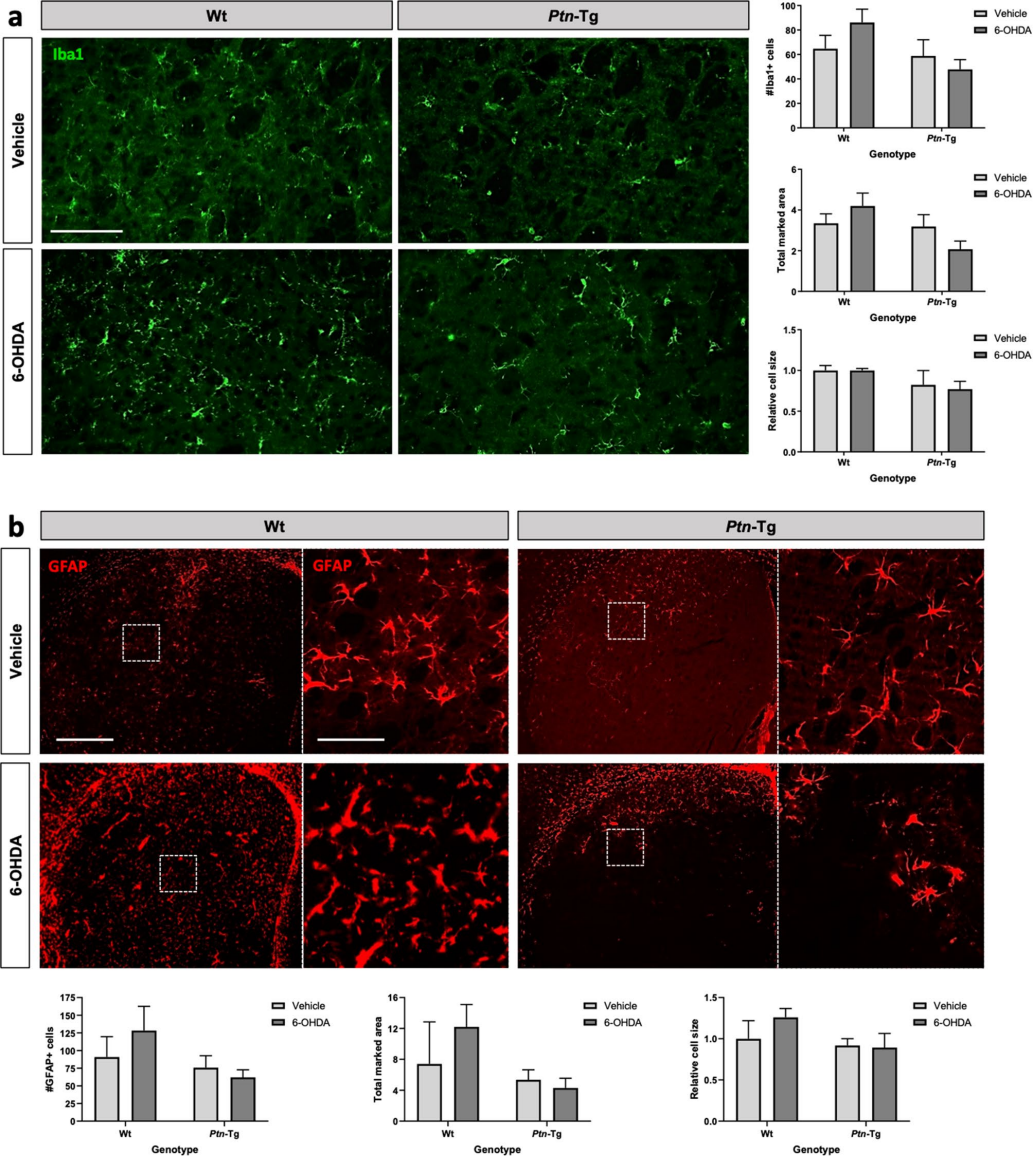
Effects of 6-OHDA on microgliosis and astrocytosis in the striatum of Wt and *Ptn*-Tg mice. Photomicrographs are representative from Iba1-immunostained (**a**, green) and GFAP-immunostained (**b**, red) striatal sections of vehicle and 6-OHDA-injected size of Wt mice and *Ptn*-Tg mice. Graphs represent quantification of data (mean ± S.E.M) obtained from the counts of Iba1+ cells, total Iba1 marked area and relative cell size (**a**) and from the counts of GFAP+ cells, total GFAP marked area and relative cell size (**b**) in the striatum. Scale bar in (**a**) = 100 μm; Scale bar in (**b**) = 500 μm (magnification image in dashed line, scale bar = 100 μm).

Taking together, the data presented here support a role of PTN against 6-OHDA-induced neurotoxicity in nigrostriatal dopaminergic circuits. Since PTN is a novel modulator of glial activation and neuroinflammation in different contexts^3,21,36,37^, we aimed to study the modulatory role of PTN overexpression on 6-OHDA-induced striatal glial responses. We tested the astrocytic and microglial response in striatal sections of vehicle- and 6-OHDA-treated Wt and *Ptn*-Tg mice. The ANOVA of the immunohistochemistry studies of the microglial marker Iba1 did not show treatment-related differences but revealed that the number of Iba1+ cells and marked area tended to be different between genotypes (Iba1+ cells, F (1,18) = 3.866, P = 0.0649; marked area, F (1,15) = 3.971, P = 0.0648) (Fig. 3a), whereas significant differences between genotypes were observed in the case of Iba1 cell size (F (1,18) = 5.577, P = 0.0297). The data reflects higher values of these three parameters in Wt mice; however, post-hoc analysis of Iba1 cell size did not reveal significant differences between individual groups. The ANOVA of the data from the immunohistochemistry studies for GFAP, an astrocyte-specific intermediate filament protein^38^, did not reveal treatment-related differences nor differences between genotypes (GFAP+ cells, F (1,14) = 2.519, P = 0.1348; marked area, F (1,13) = 3.286, P = 0.0930; cell size, F (1,14) = 2.414, P = 0.1426) (Fig. 3b).

In summary, the data suggest that PTN overexpression prevents the dopaminergic neurotoxicity induced by a low dose of 6-OHDA that does not cause alterations in motor functions or significant glial responses.

### Metabolomics analysis of *Ptn*-Tg and Wt mice

In general, the analysis performed revealed more significant metabolomic alterations associated with the genotype (*Ptn*-Tg vs. Wt) within the same treatment than between treatments (6-OHDA vs. vehicle) within the same genotype. The metabolites found significantly altered in the striatum of 6-OHDA-injected Wt mice compared with vehicle-injected Wt mice are listed in supplementary Table 1. The metabolites found significantly altered in the striatum of 6-OHDA-injected *Ptn*-Tg mice compared with vehicle-injected *Ptn*-Tg mice are listed in supplementary Table 2. These were mainly polar and small metabolites that did not belong to any particular metabolic group. Further specific analysis for small polar compounds should be carried out in order to confirm the nature of these metabolites and reveal their possible implications in this PD model.

**Figure 4:**
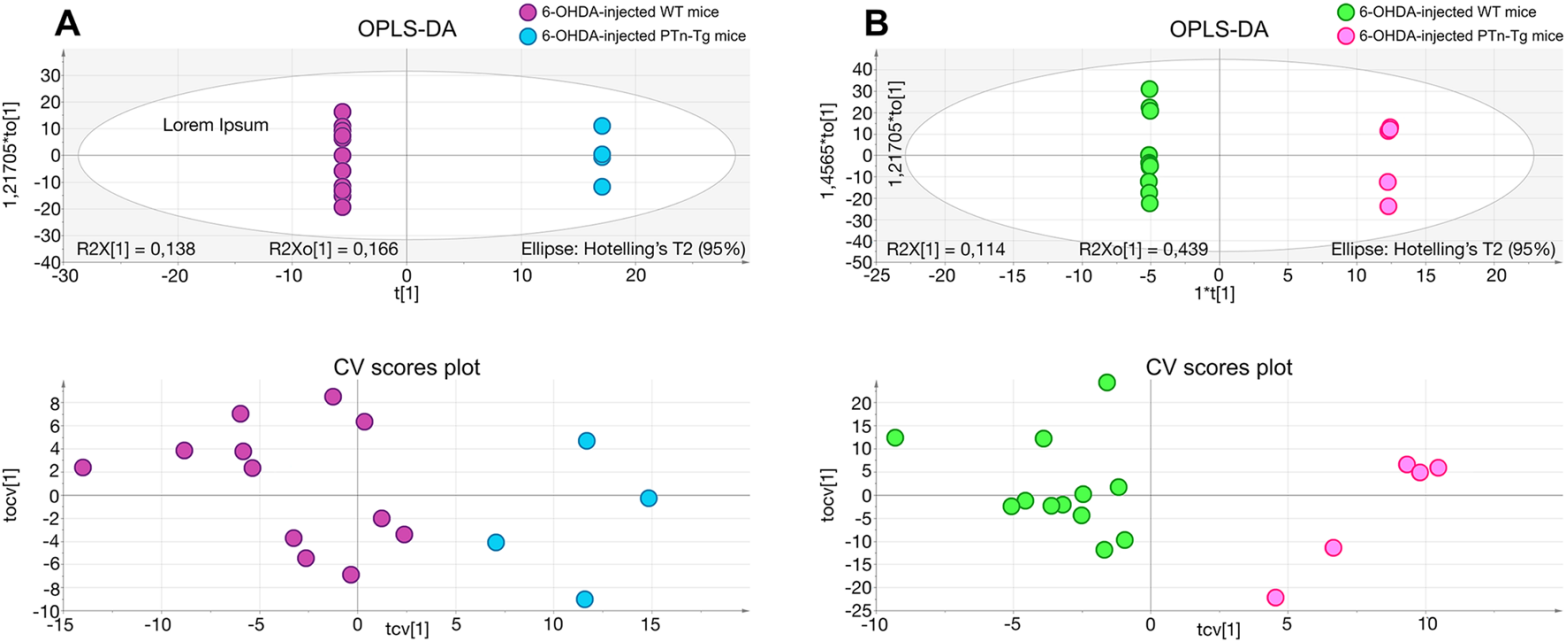
OPLS-DA regression models and cross-validated (CV) score plots showing differentiation of the metabolic profiles between 6-OHDA-injected Wt mice and 6-OHDA-injected *Ptn*-Tg mice in positive (**a**, R^2^ = 0.938, Q^2^ = 0.699) and negative (**b**, R^2^ = 0.855, Q^2^ = 0.796) ionization modes.

**Figure 5:**
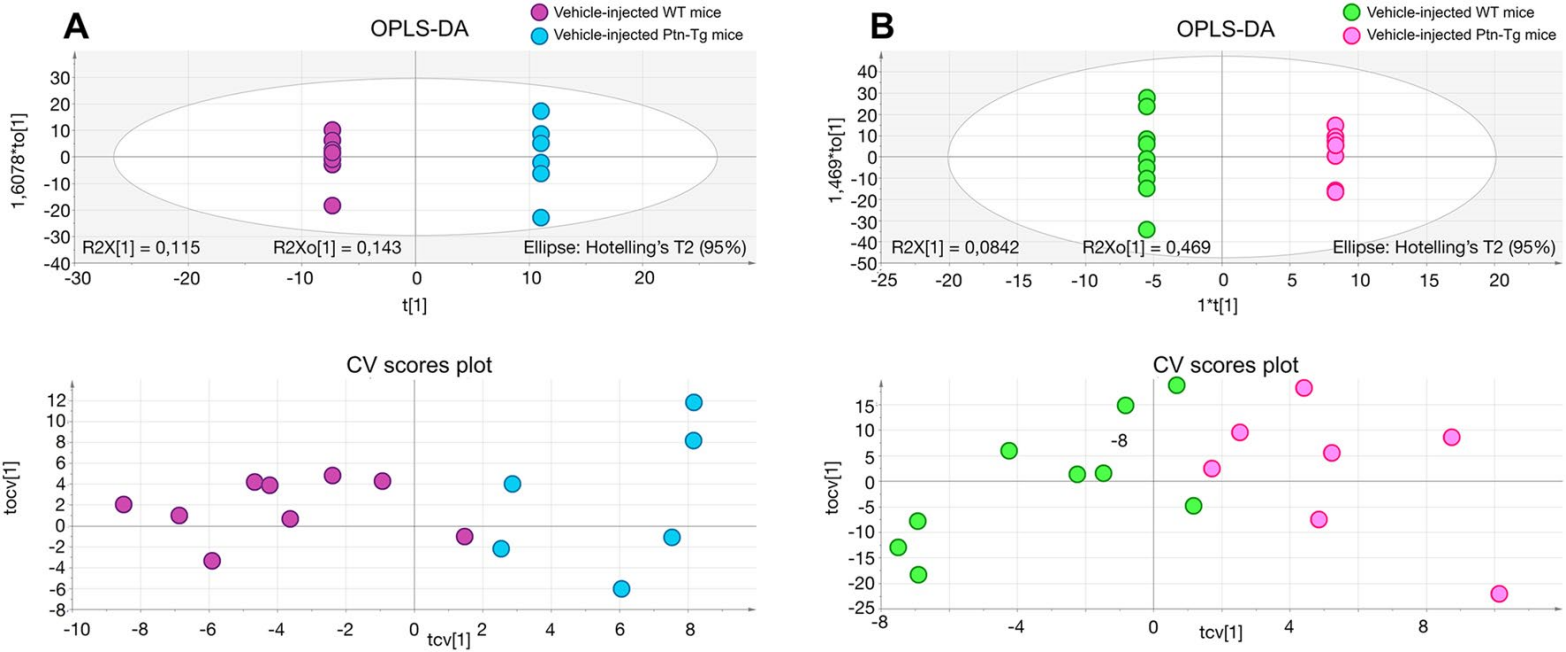
OPLS-DA regression models and cross-validated (CV) score plots showing differentiation of the metabolic profiles between vehicle-injected Wt mice and vehicle-injected *Ptn*-Tg mice in positive (**a**, R^2^ = 0.927, Q^2^ = 0.686) and negative (**b**, R^2^ = 0. 968, Q^2^ = 0.605) ionization modes.

**Figure 6:**
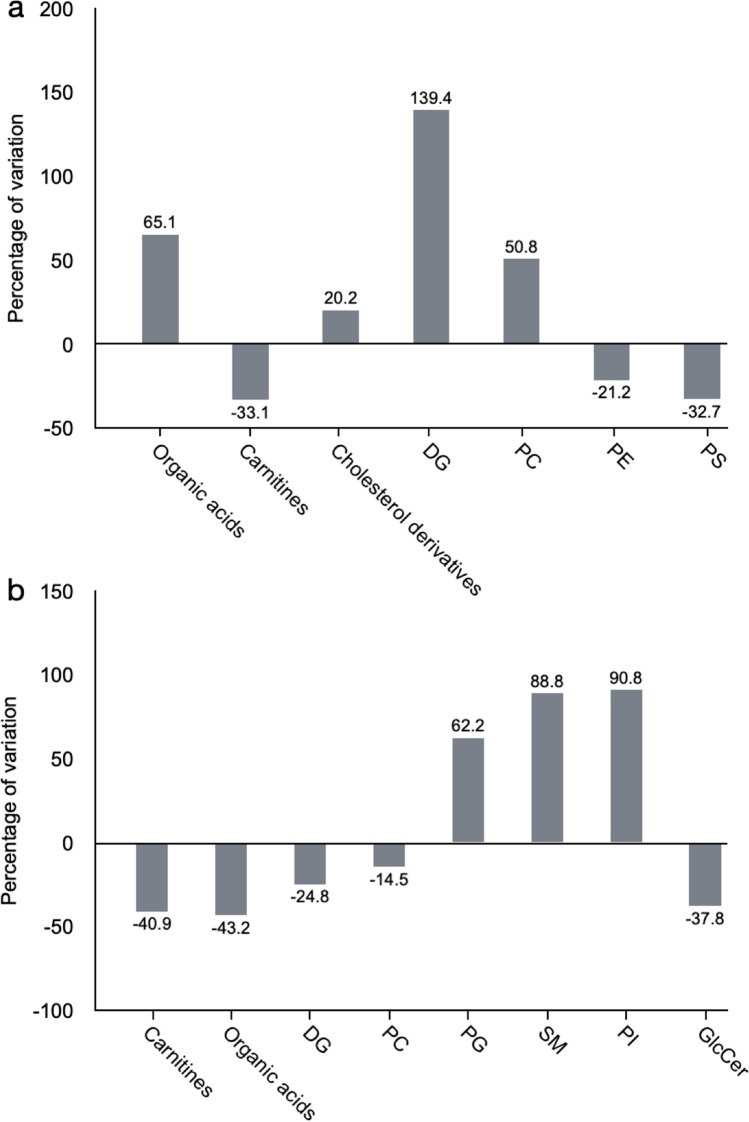
Average percentage of variation for relevant groups of metabolites changed in the different studied groups. (**a**) Percentage of variation of metabolic groups between *Ptn*-Tg mice and Wt mice injected with 6-OHDA. (**b**) percentage of variation of metabolic groups between *Ptn*-Tg mice and Wt mice injected with the vehicle. DG: Diglyceride, PC: Phosphatidylcholine, PE: Phosphatidylethanolamine, PS: Phosphatidylserine, PG: Phosphatidylglycerol, SM: Sphingomyelin, PI: Phosphatidylinositol.

In contrast, we found more profound differences between genotypes, suggesting an important role of cerebral PTN levels in the response to the dopaminergic toxic. The metabolites found significantly altered in the striatum of 6-OHDA-injected *Ptn*-Tg mice compared with 6-OHDA-injected Wt mice are listed in Table 1. Besides, Fig. 4 shows the OPLS-DA regression model together with the cross-validation scores representation, which shows how these two sets of mice are grouped according to their metabolic profiles. The metabolites found significantly altered in the striatum of vehicle-injected *Ptn*-Tg mice compared with vehicle-injected Wt mice are listed in Table 2. Similarly, Fig. 5 shows the OPLS-DA regression model together with the cross-validation scores representation, showing the grouping of the mice according to their metabolic profiles in the two models studied. Interestingly, these metabolites coordinately varied in groups (Fig. 6). The main groups of metabolites that varied between 6-OHDA-injected *Ptn*-Tg and Wt mice belonged to the groups of organic acid with relatively long chains, carnitines, cholesterol derivatives, diacylglycerols (DG), phosphatidylcholines (PC), phosphatidylethanolamines (PE) and phosphatidylserines (PS) (Fig. 6a; Table 1). The altered metabolites between vehicle-injected *Ptn*-Tg and Wt mice belonged to the groups of carnitines, groups of organic acids with relatively long chains, DG, PC, phosphatidylglycerols (PG), sphingomyelins (SM), phosphatidylinositols (PI) and glucosylceramides (GlcCer) (Fig. 6b; Table 2).

**Table 1:** Metabolites found to be significant between 6-OHDA-injected *Ptn*-Tg mice and 6-OHDA-injected Wt mice.

Feature	P-value	% VAR (*Ptn*-Tg vs Wt)	Name	Polarity	Adduct
111.9921@0.69	0.04	70.2	Methylphosphate	POS	M+H
133.0366@0.69	0.01	− 31.5	Aspartic acid	POS	M+H
197.0312@0.69	0.01	− 27.7	3-Hydroxy-2-methylpyridine-4,5-dicarboxylate	POS	M+H
133.1091@0.73	0.03	118.6	Bis (2-hydroxypropyl) amine	POS	M+H
308.2009@1.02	0.04	64.1	ent-9-L1-PhytoP	POS	M+H
279.2561@1.76	0.04	49.6	Linoleamide	POS	M+H
607.0792@0.71	0.05	− 46.7	Uridine diphosphate-N-acetylgalactosamine	NEG	M−H
147.0537@0.78	0.01	− 43.2	Glutamate/N-Acetyl-dl-serine	NEG	M−H
267.0958@0.79	0.00	− 69.2	Neuraminic acid/deoxyguanosine/adenosine	NEG	M−H
354.2078@1.01	0.04	35.4	5-hydroperoxy-7-[3,5-epidioxy-2-(2-octenyl)-cyclopentyl]-6-heptenoic acid	POS	M+H
175.0478@0.73	0.05	− 50.0	2-Amino-3-oxo-hexanedioic acid	NEG	M−H
271.2471@1.02	0.01	101.1	2-Amino-hexadecanoic acid	POS	M+H
501.3833@1.21	0.00	107.1	(Z)-2-Hexacos-17-enamidoethanesulfonic acid	POS	M+H
554.2954@1.24	0.00	131.9	2-O-(beta-d-galactopyranosyl-(1 → 6)-beta-d-galactopyranosyl) 2S-hydroxytridecanoic acid	POS	M+H
397.3196@1.35	0.01	− 34.2	O-palmitoleoylcarnitine	POS	M+H
425.3517@1.72	0.01	− 31.9	O-oleoylcarnitine/elaidic carnitine	POS	M+H
267.9405@1.05	0.01	− 21.2	1beta,3alpha,7alpha,12alpha-Tetrahydroxy-5beta-cholan-24-oic Acid	NEG	M−H
406.3028@2.31	0.01	50.0	Homochenodeoxycholic acid	POS	M+H
400.3337@2.83	0.03	47.5	24-oxocholesterol	POS	M+H
390.2775@3.12	0.02	60.1	3alpha,12alpha-Dihydroxy-5beta-chol-14-en-24-oic Acid	POS	M+H
398.351@4.32	0.03	− 35.1	22S,23S-methylenecholesterol	POS	M+H
550.4965@9.28	0.02	233.2	DG(32:1)	POS	M+H
679.5143@9.28	0.01	222.4	DG(40:9)	POS	M+NH4
583.5189@13.18	0.03	− 37.5	DG(32:1)	POS	M+NH4
1573.0315@10.31	0.03	26.4	CL(82:16)	POS	M+H
753.5314@9.82	0.00	75.8	PC(34:4)	POS	M+H
827.5451@10.62	0.03	82.9	PC(40:9)	POS	M+H
765.5453@11.46	0.00	76.9	PC(35:5)	POS	M+H
795.5775@11.6	0.01	46.6	PC(37:4)	POS	M+H
717.5687@12.55	0.00	25.6	PC(32:0)/PE(35:1)	POS	M+H
719.588@12.67	0.01	26.3	PC(32:0)/PE(35:0)	POS	M+H
689.5293@10.1	0.04	45.8	PC(O-30:1)/PE(P-33:0)	POS	M+H
691.5513@10.24	0.01	26.2	PC(30:0)/PE(33:0)	POS	M+H
777.538@11.57	0.02	53.8	PE(39:6)	POS	M+H
753.529@12.59	0.05	− 46.1	PE(37:4)	NEG	M−H
725.5306@13.5	0.02	− 72.0	PE(36:3)	NEG	M−H
879.5985@13.51	0.01	− 30.2	PE(DiMe(13,5)/DiMe(9,5))	NEG	M−H
715.5476@15.83	0.01	− 56.3	PE(35:2)	NEG	M−H
745.5509@15.33	0.00	− 35.5	PE(36:1)	NEG	M−H
632.4791@15.96	0.03	38.2	PE-Cer(32:1)	POS	M+H
1180.7394@7.89	0.00	− 81.8	Ganglioside GM3 (36:1)/NeuAcalpha2-3Galbeta1-4Glcbeta-Cer(36:1)/CL(52:2)	NEG	M−H
761.5158@10.03	0.04	− 79.1	PS(34:1)	NEG	M−H
877.5863@10.75	0.01	− 38.0	PS(43:6)	NEG	M−H
811.5324@10.76	0.04	− 36.4	PS(38:4)	NEG	M−H
877.5795@11.63	0.00	− 36.6	PS(43:6)	NEG	M−H
853.5793@11.95	0.02	− 27.1	PS(41:4)	NEG	M−H
893.6202@13.55	0.03	− 36.8	PS(44:5)	NEG	M−H
761.5308@13.81	0.02	− 37.2	PS(34:1)	NEG	M−H
833.6121@15.4	0.05	− 31.7	PS(39:0)	NEG	M−H
857.5195@9.51	0.01	28.4	PS(42:9)	POS	M+H

**Table 2:** Metabolites found to be significant between vehicle-injected *Ptn*-Tg mice and vehicle-injected Wt mice.

Feature	P-value	% VAR (*Ptn*-Tg vs Wt)	Name	Polarity	Adduct
197.0312@0.69	0.0004	− 37.6	3-Hydroxy-2-methylpyridine-4,5-dicarboxylate	POS	M+H
133.0366@0.69	0.0076	− 33.2	Aspartic acid	POS	M+H
129.0432@0.7	0.0120	− 28.7	5-Oxo-d-proline/d-pyroglutamic acid/d-5-pyrrolidone-2-carboxylic acid	POS	M+H
153.0566@0.73	0.0360	− 16.5	1,2-Diamino-4-nitrobenzene/N-dimethyl-2-aminoethylphosphonate; 2-dimethylaminoethylphosphonate	POS	M+H
279.0867@0.73	0.0360	− 36.6	Ser Ala Cys	POS	M+H
133.1091@0.73	0.0256	75.2	Bis (2-hydroxypropyl) amine	POS	M+H
183.0859@0.77	0.0016	− 45.9	Methylnoradrenaline/epinephrine/normetanephrine	POS	M+H
221.1274@0.77	0.0256	− 49.4	Dihydrozeatin	POS	M+H
102.0327@0.77	0.0028	− 39.7	Methylmalonic acid semialdehyde/2-methyl-3-oxo-propanoic acid/3-methyl pyruvic acid/Acetoacetic acid	POS	M+H
308.2009@1.02	0.0076	39.4	ent-9-L1-PhytoP	POS	M+H
257.2365@1.27	0.0076	57.0	d-erythro-sphingosine C-15	POS	M+H
365.2959@1.7	0.0120	105.9	N-Linoleoyl GABA/N-cis-octadec-9Z-enoyl-l-homoserine lactone	POS	M+H
286.2313@1.82	0.0004	− 45.1	Retinol	POS	M+H
400.3337@2.83	0.0176	43.4	(24Z),26-hydroxydesmosterol	POS	M+H
460.3251@3.63	0.0496	28.8	Stoloniferone G	POS	M+H
161.1042@0.87	0.0048	− 26.6	l-carnitine	POS	M+H
371.3066@1.25	0.0360	− 39.5	Tetradecanoylcarnitine	POS	M+H
397.3196@1.35	0.0028	− 51.9	O-palmitoleoylcarnitine	POS	M+H
447.3337@1.46	0.0016	− 40.2	O-arachidonoylcarnitine	POS	M+H
425.3517@1.72	0.0256	− 46.2	O-oleoylcarnitine/elaidic carnitine	POS	M+H
267.0958@0.79	0.0176	− 44.6	Neuraminic acid/deoxyguanosine/adenosine	NEG	M−H
398.3472@1.57	0.0360	− 34.8	Axillarenic acid/tetracosanedioic acid	POS	M+H
299.2829@1.8	0.0496	− 34.8	Amino-octadecanoic acid	POS	M+H
378.2791@1.82	0.0004	− 47.0	Norchenodeoxycholic acid	POS	M+H
312.2674@14.26	0.0016	− 40.6	10-oxo-nonadecanoic acid	POS	M+H
340.2998@17.07	0.0004	− 55.3	2-oxo-heneicosanoic acid	POS	M+H
338.2853@15.15	0.0004	− 46.9	Glycidyl oleate	POS	M+H
416.3579@2.43	0.0360	− 41.8	Amphimic acid B	POS	M+H
712.5953@10.87	0.0120	55.4	DG(43:5)	POS	M+H
640.5028@13.98	0.0360	− 21.9	DG(38:6)	POS	M+H
638.4889@14.17	0.0028	− 44.6	DG(38:7)	POS	M+H
616.5067@14.26	0.0004	− 39.2	DG(36:4)	POS	M+H
642.5181@15.15	0.0004	− 49.0	DG(38:5)	POS	M+H
644.5426@17.08	0.0004	− 44.5	DG(38:4)	POS	M+H
644.5414@18.8	0.0256	− 29.9	DG(38:4)	POS	M+H
763.5234@10.32	0.0120	14.0	PC(35:6)	POS	M+H
795.5775@11.6	0.0360	− 27.4	PC(37:4)	POS	M+H
809.596@12.95	0.0028	− 12.5	PC(38:4)	POS	M+H
773.5914@13.23	0.0360	− 31.2	PC(35:1)	POS	M+H
813.6275@15.27	0.0496	− 15.2	PC(38:2)	POS	M+H
907.6355@14.85	0.0076	− 37.5	PE-NMe(11D5/13M5)	NEG	M−H
776.6005@11.68	0.0120	68.9	PG(37:0)	NEG	M−H
886.5582@9.31	0.0004	55.5	PGP(38:0)	POS	M+H
728.5879@9.48	0.0028	135.6	SM(36:2)	POS	M+H
730.6061@10.89	0.0120	70.1	SM(36:1)	POS	M+H
744.6081@12.3	0.0076	60.6	SM(37:1)/PE-Cer(40:1)	POS	M+H
882.5225@7.58	0.0360	155.4	PI(38:6)	NEG	M−H
858.5229@7.78	0.0256	60.3	PI(36:4)	NEG	M−H
884.5382@8.4	0.0120	133.0	PI(38:5)	NEG	M−H
886.5517@9.92	0.0120	45.6	PI(38:4)	NEG	M−H
954.5383@9.93	0.0028	59.6	PI(44:12)	NEG	M−H
811.6861@19.58	0.0496	− 26.9	GlcCer(42:1)	NEG	M−H
813.7112@20.44	0.0256	− 48.7	GlcCer(42:0)	NEG	M−H
1180.7394@7.89	0.0028	− 61.5	Ganglioside GM3 (36:1)	NEG	M−H

The metabolomics data has been deposited to the Metabolomics Workbench repository (available at https://www.metabolomicsworkbench.org) with the dataset identifier ST001810.

## Discussion

Previous studies supported that PTN exerts neurotrophic effects on dopaminergic neurons, suggesting an important role of this cytokine in PD^39^. Previously, it was shown that striatal and nigral PTN over-expression provides neuroprotection against 6-OHDA in rats^16,17^. This effect was extended in the present work by uncovering the correlation between the effects caused by PTN over-expression in the 6-OHDA-injured mouse striatum with relevant changes in striatal phosphatidylcholines, phosphatidylethanolamines, phosphatidylserines, phosphatidylinositols and diglycerides.

Assessing the efficacy of neuroprotective strategies in 6-OHDA models with a full (> 90% striatal dopaminergic denervation) or partial (60–70%) lesion is less than ideal^40^, probably because of the severity of the injury and the high variability of the resulting symptoms. Here, using a low dose of 6-OHDA (5 µg) to cause a partial lesion in mice, we show that the striatal dopaminergic denervation caused by 6-OHDA is prevented in mice with transgenic PTN overexpression in the brain. In agreement with previous studies with similar levels of striatal denervation^41,42^, the TH loss caused by this dose of 6-OHDA in Wt mice did not cause significant motor alterations. Moreover, 6-OHDA-induced loss of dopaminergic cells in the substantia nigra seems to be reduced in *Ptn*-Tg mice compared to Wt mice. It has to be noted that we observed a slightly reduced number of TH+ cells in the substantia nigra of vehicle-injected *Ptn*-Tg mice compared to Wt mice. Thus, we cannot rule out the possibility that the effect of 6-OHDA is partially masked in *Ptn*-Tg mice when compared with vehicle-injected *Ptn*-Tg mice. Although the decrease in TH+ cells in the SNpc is widely considered as dopaminergic neurons loss^43,44^, in some cases features of apoptosis or necrosis have not been detected^45,46^, suggesting that lack of TH does not necessarily imply cell death. These differences may be related with the dose and timeline of the study. Therefore, it cannot be assumed that the data presented here represent survival of dopaminergic neurons. However, based on the known neurotrophic actions of PTN on dopaminergic neurons^4,17,47,48^, it seems reasonable to suggest a neuroprotective role of this cytokine in the SN. Overall, the data demonstrate that PTN limits the nigrostriatal dopaminergic injury in this mouse model of PD.

Striatal gliosis is a hallmark of PD and, in general, of the neuroimmune response triggered by neurotoxins such as 6-OHDA. Besides, PTN has been recently shown to modulate these glial responses after different insults including LPS^21,37^ and amphetamine^6,36^ administrations. Thus, we aimed to test if the protective effects of PTN on dopaminergic neurons are paralleled by a regulation of potential glial responses after the administration of a low dose of 6-OHDA. We did not observe relevant responses of microglia and astrocytes to the striatal injection of 6-OHDA in either genotype, suggesting that the striatal TH loss was not accompanied by generalized glial responses. It is important to note that a limitation of this study is that glial responses were only assessed in the striatum to be able to correlate these responses with potential changes in the striatal metabolome. However, testing the neuroinflammatory response in the SN in this experimental model should be of interest for future studies. Another limitation is that we only assessed nigrostriatal injury and glial responses at one time point, ten days after 6-OHDA intrastriatal injection. Thus, it is possible that TH loss or delayed glial changes are more pronounced at a later time point.

The cause of striatal degeneration of dopaminergic terminals is not well known, but increasing evidence suggests a differential astrocytes participation in this process among other factors. Astrocytes can promote or prevent neuronal damage, and the loss of the balance between both opposing actions could be critical for the onset and progression of PD^49–52^. The use of suitable animal models could enhance the understanding of the role of striatal denervation and astrocytosis in PD. Thus, in order to improve our understanding of altered metabolic pathways and metabolites in PD, we used the *Ptn*-Tg mouse as a genetic model of reduced 6-OHDA-induced striatal denervation compared to control, Wt mice. We hypothesize that data from metabolomics studies using this model could result in novel treatments for alterations of metabolic pathways in PD and/or could be used for disease prediction and early diagnosis since our model was performed in the asymptomatic phase.

This study reveals interesting metabolites that significantly discriminate the more injured 6-OHDA-injected Wt striatum and the more protected 6-OHDA-injected *Ptn*-Tg striatum. Notably, we detected groups of metabolites, mostly corresponding to phospholipids, together with a wide variety of organic acids and carnitines, whose trends were opposite in both groups (Fig. 6a). The fact that entire groups of phospholipids tend to vary together is very significant because α-Synuclein is a lipid-binding protein that interacts with phospholipids and fatty acids^53,54^.

We found increased levels of 8 forms of phosphatidylcholine (PC) in the striatum of 6-OHDA-injected *Ptn*-Tg mice compared to Wt mice. Interestingly, this pattern was opposite in vehicle-injected mice. PC is the most abundant phospholipid in cellular membranes^55^. Decreased levels of PC containing polyunsaturated fatty acyl side chains have been shown in PD brains^56–58^. Importantly, injection of 6-OHDA in rats causes decreases in most PC species in the nigrostriatal circuits^59^. Our data point to a role of PTN in the increase of PC species after 6-OHDA injection to *Ptn*-Tg mice compared to Wt mice, which correlated with the significant reduction of dopaminergic injury in their striata. Since PC is known to be involved in neuronal differentiation, neurite outgrowth, and axonal elongation^60^, it is tempting to hypothesize that the neurotrophic effects of PTN on dopaminergic neurons can be mediated, at least partially, by the increase of PC species.

After PC, the next most abundant group of phospholipids is the group of phosphatidylethanolamines (PE). Five PE species were found decreased in the striatum of 6-OHDA-injected *Ptn*-Tg mice compared to Wt mice, whereas two species of PE were increased. Decreases in multiple PE species were observed in brains of PD patients^56,61^. In contrast, increased PE synthesis in the substantia nigra of PD patients has been suggested during the course of PD development^62^. In general, increased PE synthesis contributes to the rescue of neurons^63,64^. Our data suggest that some PE (such as PE (39:6) and PE-Cer (32:1)) might be critical in the reported neuroprotective effects of this phospholipid since they are the only PE increased in the striatum of the less vulnerable genotype to 6-OHDA effects, the *Ptn*-Tg mouse, while the major trend of the majority of these lipids is to decrease in the 6-OHDA-injected *Ptn*-Tg mice compared to Wt mice.

Eight species of phosphatidylserines (PS) were found decreased in the striatum of 6-OHDA-injected *Ptn*-Tg mice compared to Wt mice, whereas only one was increased. Decreased PS levels have been observed in 6-OHDA-treated SH-SY5Y cells^65^, a cellular model of PD, and in plasma from PD patients^66^. PS is a less abundant membrane phospholipid but is a relevant precursor of mitochondrial PE. Accordingly, as it happened with PE, PS increases and elevated activity of phosphatidylserine synthase, the enzyme responsible for PS synthesis, have been found in the SN of PD patients^62^. Our results support this hypothesis since general decreases of PS species were found in the less vulnerable genotype to 6-OHDA-induced striatal dopaminergic injury.

Five species of phosphatidylinositols (PI) were found increased in the striatum of vehicle-injected *Ptn*-Tg mice compared to Wt mice, suggesting that increased PTN brain levels correlate with increased PI levels in basal conditions. In contrast, we did not find significant changes in the striatum of 6-OHDA-injected *Ptn*-Tg mice compared to Wt mice. The PI total lipid class is significantly reduced in the SN of PD patients^58^, suggesting an implication of PI depletion in PD. Our data support the possibility that the increases of PI species found in basal conditions in *Ptn*-Tg mice may play a role in the lower dopaminergic injury found in this genotype after 6-OHDA insult.

Six species of diglycerides (DG) were decreased in the striatum of vehicle-injected *Ptn*-Tg mice compared to Wt mice, whereas only one was increased. In contrast, we found increases of two DG species in the striatum of 6-OHDA-injected *Ptn*-Tg mice compared to Wt mice, whereas only one was found decreased. The relevance of these changes needs to be clarified. However, variations in DG species were expected and validate our studies because DGKQ, a diacylglycerol kinase controlling cellular DG, is a designated PD risk factor^67,68^.

Our data confirm previous findings of known phospholipids that are associated to neurodegeneration, as observed in animal models of PD. Besides, we provide new evidence supporting that the potentiation of PTN signaling may be a novel target for PD, particularly in early stages of the disease since our model was performed in an asymptomatic phase. New lipid-related PD drug candidates emerge from this study, which suggests their significant role in PD. The data presented here support the increasingly recognized “lipid cascade” in PD.

## Supplementary Information

Supplementary Figure 1.

Supplementary Table 1.

Supplementary Table 2.

## Data Availability

The datasets used and/or analysed during the current study available from the corresponding author on reasonable request.
