# Supplementary material for: Metabolomics and biochemical alterations caused by pleiotrophin in the 6-hydroxydopamine mouse model of Parkinson’s disease
Source: Sci Rep. 2022 Mar 4;12:3577. doi: 10.1038/s41598-022-07419-6 (PMC8897456; doi:10.1038/s41598-022-07419-6)
Supplement: Supplementary file 1 — Supplementary Figure 1. [file 41598_2022_7419_MOESM1_ESM.docx]

**SUPPLEMENTARY DATA**


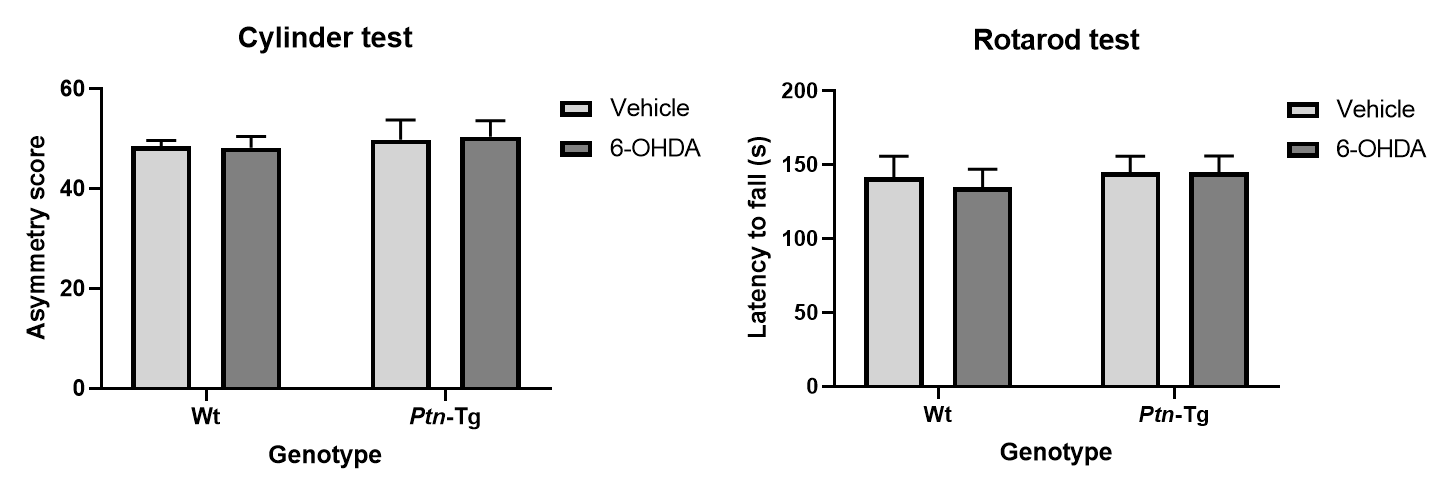


**Figure S1. Normal motor coordination abilities revealed by the cylinder test and rotarod test in Wt and *Ptn*-Tg mice**. Asymmetry score in the cylinder test reflects the percentage of left limb contacts as a percentage of the total performance (a). Latency to fall during an accelerating rotarod test. The maximum time standing on the rod of four attempts is represented (b). Data are presented as mean ± SEM.
