# Supplementary material for: Metabolomics and biochemical alterations caused by pleiotrophin in the 6-hydroxydopamine mouse model of Parkinson’s disease
Source: Sci Rep. 2022 Mar 4;12:3577. doi: 10.1038/s41598-022-07419-6 (PMC8897456; doi:10.1038/s41598-022-07419-6)
Supplement: Supplementary file 2 — Supplementary Table 1. [file 41598_2022_7419_MOESM2_ESM.docx]

| **Feature** | **p-value** | **% VAR Wt (6-OHDA vs VEH)** | **Name** | **Polarity** | **Adduct** |
| --- | --- | --- | --- | --- | --- |
| 257.1041@0.89 | 0,028 | -25,7 | Mefenamic acid Metabolite (Anthranilic acid, N-(a3-hydroxy-2,3-xylyl)-) | POS | M+H |
| 345.3236@1.79 | 0,009 | 51,9 | Hydroxy-eicosanoic acid | POS | M+NH4 |
| 299.2829@1.8 | 0,028 | -27,7 | 12-amino-octadecanoic acid | POS | M+H |
| 362.2849@2.72 | 0,018 | -33,1 | Norlithocholic acid | POS | M+H |
| 402.2807@1.78 | 0,004 | -24,6 | 1alpha-hydroxy-26,27-dinorcholecalciferol 25-carboxylic acid; 7b-Hydroxy-3-oxo-5b-cholanoic acid | POS | M+H |
| 378.2791@1.82 | 0,000 | -39,4 | Norchenodeoxycholic acid; Nordeoxycholic acid; Norhyodeoxycholic acid | POS | M+H |
| 286.2313@1.82 | 0,000 | -38,9 | Retinol | POS | M+H |
| 283.2882@2.56 | 0,012 | 46,2 | Stearamide | POS | M+H |
| 317.2923@1.37 | 0,012 | 41,4 | Phytosphingosine | POS | M+H |
| 358.3081@9.32 | 0,000 | 72,6 | MG(18:0) | POS | M+H |
| 360.2671@17.06 | 0,007 | -33,2 | MG(20:4) | POS | M+H-H2O |
| 340.2998@17.07 | 0,003 | -37,5 | MG(18:0) | POS | M+H-H2O |
| 598.5003@7.36 | 0,002 | 56,9 | DG(26:4) | POS | M+H-H2O |
| 624.5168@7.97 | 0,004 | 54,5 | DG(38:5) | POS | M+H-H2O |
| 644.5426@17.08 | 0,002 | -31,8 | DG(38:4) | POS | M+H |
| 616.5067@14.26 | 0,007 | -25,5 | DG(36:4) | POS | M+H |
| 624.5144@15.17 | 0,007 | -38,8 | DG(38:5) | POS | M+Na |
| 728.5879@9.48 | 0,012 | 56,4 | SM(36:2); PE-Cer(d39:2) | POS | M+H |
| 795.5775@11.6 | 0,028 | -27,6 | PC(37:4) | POS | M+H |
| 863.6431@16.29 | 0,041 | -26,4 | PC(42:5) | POS | M+H |

Supplementary Table 1. Metabolites found to be significant between 6-OHDA-injected Wt mice and vehicle (VEH)-injected Wt mice.
