# Supplementary material for: Metabolomics and biochemical alterations caused by pleiotrophin in the 6-hydroxydopamine mouse model of Parkinson’s disease
Source: Sci Rep. 2022 Mar 4;12:3577. doi: 10.1038/s41598-022-07419-6 (PMC8897456; doi:10.1038/s41598-022-07419-6)
Supplement: Supplementary file 3 — Supplementary Table 2. [file 41598_2022_7419_MOESM3_ESM.docx]

Supplementary Table 2. Metabolites found to be significant between 6-OHDA-injected *Ptn*-Tg mice and vehicle (VEH)-injected *Ptn*-Tg mice.

| **Feature** | **p-value** | **% VAR *Ptn*-Tg (6-OHDA vs VEH)** | **Name** | **Polarity** | **Adduct** |
| --- | --- | --- | --- | --- | --- |
| 131.0698@0.71 | 0.038 | 33.1 | Creatine | POS | M+H |
| 370.2196@0.75 | 0.038 | 116.3 | Octaethylene glycol | POS | M+H |
| 434.2103@0.91 | 0.010 | 67.1 | 4,5-Dihydro-drospirenone-3-sulfate | POS | M+H |
| 579.2907@2.3 | 0.017 | -75.9 | Fumitremorgin A; p-Hydroxyfosinopril | NEG | M-H |
| 617.2908@2.31 | 0.030 | -45.3 | Kinetensin 4-7 | NEG | M+FA-H |
| 378.1462@0.92 | 0.010 | 50.0 | 6,11-Dihydroxy-3-methyl-3-(4-methyl-3-pentenyl)-3H,7H-pyrano[2,3-c]xanthen-7-one; Uvaretin; Demethylcalabaxanthone | POS | M+H |
| 428.2334@39.28 | 0.019 | 28.9 | Irbesartan; Aspidoalbine | POS | M+H |
| 782.0658@12.22 | 0.019 | 112.8 | Emblicanin A; Punicalin | POS | M+H |
| 450.2054@0.92 | 0.019 | 46.9 | Glabrescione B | POS | M+H |
| 683.1973@15.9 | 0.038 | 15.6 | 3''-Adenylylspectinomycin | POS | M+Na |
| 685.195@15.91 | 0.019 | 15.6 | Okanin 4-methyl ether 4'-O-(2''-O-caffeoyl-6''-O-acetylglucoside) | POS | M+NH4 |
| 757.2165@19.37 | 0.010 | 53.8 | Cyanidin 3-rutinoside-5-glucoside | POS | M+H |
| 190.136@2.42 | 0.038 | 50.7 | trans-beta-damascenone; 2-(3-Phenylpropyl)tetrahydrofuran | POS | M+H |
| 110.0727@2.42 | 0.019 | 67.9 | 2E,4Z-Heptadienal; 2-Isopropylfuran | POS | M+H |
| 361.2959@2.42 | 0.038 | 62.2 | N-(1R-methyl-2-hydroxy-ethyl) arachidonoyl amine | POS | M+H |
| 243.1841@2.42 | 0.019 | 56.4 | N-Undecanoylglycine | POS | M+H |
| 248.1981@0.95 | 0.038 | 33.4 | alpha-Butyl-omega-hydroxypoly(oxyethylene) poly(oxypropylene) | POS | M+H |
| 318.2773@1.08 | 0.038 | 60.4 | 1-O-(2R-hydroxy-pentadecyl)-sn-glycerol | POS | M+H |
| 267.099@1.1 | 0.038 | -51.2 | Prinomide; Adenosine; Vidarabine | POS | M+H |
| 280.206@1.14 | 0.038 | 39.6 | 12S-hydroxy-5Z,8E,10E-heptadecatrienoic acid; 3-Methyl-5-pentyl-2-furanheptanoic acid; Valeracetate | POS | M+H |
| 199.0621@0.79 | 0.019 | 51.8 | Isodictamnine | POS | M+H |
| 297.172@0.83 | 0.038 | 72.8 | 10,11-Dihydro-10,11-dihydroxyprotriptyline | POS | M+H |
| 125.0146@0.73 | 0.010 | 61.6 | Taurine | POS | M+H |
| 177.1252@0.73 | 0.010 | 161.1 | Bethanidine | POS | M+H |
| 496.2289@0.75 | 0.019 | 142.0 | Glaucarubin | POS | M+H |
| 326.1981@0.75 | 0.038 | 61.7 | Hydroquinine; Hydroquinidine; Tortuosamine | POS | M+H |
| 1161.3516@12.16 | 0.019 | -68.6 | (9Z,12Z,15Z,18Z)-3-oxotetracosatetraenoyl-CoA | POS | M+K |
| 452.2036@0.75 | 0.019 | 102.8 | 8-Butanoylneosolaniol; Tyr Asp Arg | POS | M+H |
| 213.2455@1 | 0.038 | 45.1 | Tetradecylamine | POS | M+H |
| 586.508@4.66 | 0.019 | -63.5 | Heneicosanyl palmitoleate | POS | M+K |
| 362.2849@2.72 | 0.010 | -51.2 | 17-(4-Hydroxyphenyl)heptadecanoate | POS | M+H |
| 338.2853@15.15 | 0.038 | -22.5 | Glycidyl oleate | POS | M+H |
| 343.311@1.5 | 0.010 | 96.2 | Ethanolamine Oleate | POS | M+H |
| 345.3236@1.79 | 0.019 | 113.5 | 3R-hydroxy-eicosanoic acid | POS | M+NH4 |
| 362.2849@2.72 | 0.010 | -51.2 | Norlithocholic acid | POS | M+H |
| 390.2775@3.12 | 0.019 | 65.7 | 3alpha,12alpha-Dihydroxy-5beta-chol-14-en-24-oic Acid | POS | M+H |
| 255.2565@1.87 | 0.038 | 53.7 | Palmitamide | POS | M+H |
| 525.4141@1.71 | 0.019 | 258.6 | N-n-Butyl-N-methyl-11-[3,17beta-dihydroxyestra-1,3,5(10)-trien-7alpha-yl]undecanamide | POS | M+H |
| 283.2882@2.56 | 0.010 | 41.9 | Stearamide | POS | M+H |
| 279.2561@1.76 | 0.010 | 89.6 | Linoleamide | POS | M+H |
| 161.1042@0.87 | 0.010 | 52.8 | L-carnitine | POS | M+H |
| 416.3579@2.43 | 0.010 | 112.6 | Palmitoylcarnitine | POS | M+NH4 |
| 769.5925@14.18 | 0.010 | -21.3 | PC(P-36:2) | POS | M+H |
| 837.6262@14.99 | 0.010 | -19.2 | PC(40:4) | POS | M+H |
| 863.6431@16.29 | 0.019 | -34.3 | PC(42:5) | POS | M+H |
| 757.5622@10.27 | 0.019 | 32.9 | PC(34:2) | POS | M+H |
| 817.5652@9.78 | 0.038 | 47.1 | PC(39:7) | POS | M+H |
| 691.5513@10.24 | 0.019 | 28.3 | PC(O-30:0) | POS | M+H |
| 795.5775@11.6 | 0.038 | 46.2 | PC(37:4) | POS | M+H |
| 853.5605@10.82 | 0.019 | 50.6 | PE-NMe(44:10) | POS | M+H |
| 777.538@11.57 | 0.019 | 58.1 | PE(39:6); PE-NMe(38:6) | POS | M+H |
| 827.5451@10.62 | 0.019 | 105.8 | PE-NMe(42:9) | POS | M+H |
| 777.5638@14.67 | 0.038 | -25.6 | PE(40:6) | POS | M+H |
| 789.5425@12.28 | 0.009 | -83.1 | PE(38:4) | NEG | M+Cl |
| 753.5314@9.82 | 0.038 | 54.6 | PE-NMe(36:4); PE-NMe(36:4) | POS | M+H |
| 614.4923@14.13 | 0.010 | 117.7 | DG(36:5) | POS | M+H |
| 638.4889@14.17 | 0.010 | 78.3 | DG(38:7) | POS | M+H |
| 624.5144@15.17 | 0.038 | -31.6 | DG(38:5) | POS | M+H-H2O |
| 662.5092@15.05 | 0.030 | -53.4 | DG(26:4) | NEG | M+FA-H |
| 632.4791@15.96 | 0.010 | 44.6 | PA(P-32:0) | POS | M+H |
| 682.4961@17.09 | 0.010 | 31.7 | PA(O-36:4) | POS | M+H |
| 702.5727@9.35 | 0.038 | -71.8 | SM(d34:1); PE-Cer(37:1) | POS | M+H |
| 864.5692@11.35 | 0.030 | -43.5 | PI(36:1) | NEG | M-H |
| 797.5403@12.33 | 0.019 | 107.7 | PI-Cer(34:0) | POS | M+H |
| 776.6005@11.68 | 0.030 | -39.7 | PG(P-37:0) | NEG | M-H |
| 877.5863@10.75 | 0.017 | -44.7 | PS(43:6) | NEG | M-H |
| 569.3667@2.35 | 0.030 | -52.3 | 1-(2-methoxy-eicosanyl)-sn-glycero-3-phosphoserine | NEG | M-H |
| 833.6102@15.4 | 0.030 | -32.8 | PS(39:0) | NEG | M-H |
| 857.5195@9.51 | 0.019 | 21.8 | PS(42:9) | POS | M+H |
| 829.576@12.02 | 0.017 | 41.5 | PS(39:2) | NEG | M-H |
| 2128.0623@4.85 | 0.009 | -45.5 | NeuAcalpha2-3Galbeta1-3(NeuAcalpha2-6)GalNAcbeta1-4(NeuAcalpha2-3)Galbeta1-4Glcbeta-Cer(d36:1) | NEG | M-H |
| 565.5471@5.25 | 0.019 | 28.8 | N-Stearoylsphingosine; Cer(d36:1) | POS | M+H |
| 537.5159@10.03 | 0.038 | 53.9 | Cer(d34:1) | POS | M+H |
